# A decision aid regarding long-term tube feeding targeting substitute decision makers for cognitively impaired older persons in Japan: A small-scale before-and-after study

**DOI:** 10.1186/1471-2318-14-16

**Published:** 2014-02-05

**Authors:** Yumiko Kuraoka, Kazuhiro Nakayama

**Affiliations:** 1 Department of Nursing, St. Luke’s College of Nursing, 10-1 Akashi-cho, Chuo-ku, Tokyo 104-0044, Japan

**Keywords:** Decision-making, Feeding tube, Gastrostomy, Older people

## Abstract

**Background:**

In Japan, there is no decision-making guide regarding long-term tube feeding that specifically targets individuals making decisions on behalf of cognitively impaired older persons (substitute decision makers). The objective of this study was to describe the development and evaluation of such a decision aid.

**Methods:**

In this before-and-after study, participants comprised substitute decision makers for 13 cognitively impaired inpatients aged 65 years and older who were being considered for placement of a percutaneous endoscopic gastrostomy tube in acute care hospitals and mixed-care hospitals in Japan. Questionnaires were used to compare substitute decision makers’ knowledge, decisional conflict, and predisposition regarding feeding tube placement before and after exposure to a decision aid. The acceptability of the decision aid was also assessed. Paired *t*-tests were used to compare participants’ knowledge and decisional conflict scores before and after using the decision aid.

**Results:**

Substitute decision makers showed significantly increased knowledge (P < .001) and decreased decisional conflict (P < .01) regarding long-term tube feeding after using the decision aid. All substitute decision makers found the decision aid helpful and acceptable.

**Conclusions:**

The decision aid facilitated the decision-making process of substitute decision makers by decreasing decisional conflict and increasing knowledge.

## Background

In Japan, the percentage of the population aged 65 years and over was 23.3% in 2011 [1], and those who have swallowing difficulty are increasing in number. In many patients, percutaneous endoscopic gastrostomy (PEG) is performed and tube feeding is used for artificial nutrition. A private-sector research institution in Japan [2] reported that PEG kit sales exceeded 100,000 units in 2005, and more than 700,000 people exchanged PEG kits in 2010. In the United States and Europe, insertion of feeding tubes is not typically performed to prolong life, prevent aspiration pneumonia, heal pressure ulcers, or improve quality of life [3,4]. Several studies have investigated the probability of survival of elderly patients in whom feeding tubes were placed. One study showed that the probability of surviving 1.5 years after referral for PEG was 35% among 97 residents [5]. Another study showed that overall 1-year mortality was 62% among 149 hospitalized patients [6]. In Japan, a survey of survival of geriatric patients after PEG [7] found that 99%, 95%, 88%, 75%, and 66% of 931 patients survived more than 7 days, 30 days, 60 days, 6 months, and 1 year, respectively. Of the same 931 patients, 50% and 25% of those who had feeding tubes survived 753 and 1647 days, respectively. Although some patients in that study had dementia (15% had severe dementia, 3% had mild dementia, 14% had other types of dementia), prognosis did not differ between patients with and without dementia [7]. A previous study on the perception of families of dementia patients who underwent PEG reported that 26.7% of 33 families answered that they wavered in their judgment of PEG between “good” and “not good”, and 13.3% answered “not good” [8]. In Japan, more than half of geriatric patients with PEG survive longer than 2 years. However, the quality of life of these patients is often poor. Since elderly patients with cognitive impairment often cannot make choices for themselves, a family member must make the choice on the patient’s behalf in many cases. For such family members acting as substitute decision makers, the decision often involves many conflicting facts and emotions and creates a heavy mental burden.

Few studies have examined decision-making in the use of feeding tubes [9-11]. A survey of five states in the USA found that 13.7% of 486 family members stated that there was no discussion about feeding tube insertion, and 41.6% reported having a discussion shorter than 15 minutes. The risks associated with feeding tube insertion were not discussed in one-third of the cases, and 51.8% felt that the healthcare provider was strongly in favor of feeding tube insertion [9]. Thus, in deciding whether to implement tube feeding, communication between the patient’s family and healthcare providers is often brief. Another study in Japan involving cognitively impaired elderly persons at home who underwent PEG reported that the person himself/herself was not asked about PEG, but that the treatment choice reflected the family’s own feelings; that decision-making can involve both positive consent and negative consent; and that the family’s manpower and the physician’s explanations have an influence on decision-making [10]. A survey of 4506 physicians who were members of the Japan Geriatrics Society found that only 6% stated that they did not have difficulty determining whether artificial hydration and nutrition should be started, and half stated that an ethical problem arises when deciding to withhold artificial hydration and nutrition [11]. In Japan, since families and physicians often consider cognitively impaired older patients to be unable to make their own decisions, physicians are generally relied upon for decisions involving artificial nutrition. However, because—just like family members—healthcare providers involved in determining the appropriateness of tube feeding frequently experience difficulty in making a decision, it is very important that information regarding the principles of substitute decision-making and PEG be presented to the decision maker. In order to ensure that the family can understand the situation and make an educated decision, it is necessary for the physician to provide detailed information about PEG.

The Japan Geriatrics Society has developed guidelines for decision-making processes in elderly care, focusing on indications for artificial hydration and nutrition [12]. The guidelines include statements about the decision-making process in medical treatment and care, about life and its value, and about the importance of the selection of artificial hydration and nutrition and when to reduce their quantity or to stop them altogether. However, these guidelines were created for healthcare providers, and they are not easy to use for patients or family members. The Tokyo University Thanatology/Applied Ethics Center created a decision-making “process note” for patients and families who must decide whether to initiate artificial hydration and nutrition [13]. This “process note” provides information about not only tube feeding but also vein injection and other processes. In Japan, however, there is no such decision-making guide for tube feeding that specifically targets substitute decision makers.

A tube feeding decision aid booklet for substitute decision makers was designed at the Ottawa Health Research Institute, and is available to the public on their homepage. The usefulness of this booklet has been reported [14]. The present report describes the development of a Japanese version of this decision aid and its evaluation.

## Methods

This study complied with the Declaration of Helsinki and was approved by the Research Ethics Committee of St. Luke’s College of Nursing (approval number: 11-010).

### Development of the decision aid

A tube feeding decision aid designed at the Ottawa Health Research Institute was specifically prepared for a substitute decision maker who must decide whether to allow placement of a PEG tube in a cognitively impaired person 65 years or older who is unable to eat independently [15]. The decision aid contains information about the following areas: common causes of eating and swallowing problems in older persons with cognitive impairment, such as damage to the muscles and nerves needed for proper swallowing (e.g., stroke), inability to eat independently (e.g., Alzheimer’s disease), blockage of the esophagus (e.g., esophageal cancer), or severe loss of appetite or interest in eating (e.g., major depression); technical considerations regarding the placement and use of PEG tubes; principles of substitute decision-making; the risks and benefits of tube feeding; the option of supportive care; and some considerations regarding future discontinuation of PEG tube feeding if the substitute decision maker opts for the intervention. Various panels were involved in creating, reviewing, and evaluating the decision aid.

The Japanese version was developed as follows. First, the developer’s consent was obtained, and the tube feeding decision aid booklet was translated into Japanese. Since an aid for substitute decision makers must be easy to read, the Japanese was refined. Next, the data on the probability of survival in European and American people were adapted to be appropriate for Japanese people. The original version of the decision aid included a chart showing that 100%, 80%, 75%, 55%, 45% of 100 elderly patients who have feeding tubes will be alive after 1 day, 30 days, 60 days, 6 months, and 1 year. We did not include this chart in the Japanese version of the decision aid. Instead, we used the results of a previous study in Japan [7] and included a diagram to explain data on the probability of survival (99%, 95%, 88%, 75%, and 66% of 931 patients survived more than 7 days, 30 days, 60 days, 6 months, and 1 year, respectively, and 50% and 25% of 931 elderly patients were alive after 753 and 1647 days, respectively). Four physicians were involved in creating the prototype decision aid. Two physicians recommended that the principles of substitute decision-making be moved to Chapter 1 from Chapter 3, and one physician recommended adding data of quality of life of patients who have undergone PEG. Thus, we moved the principles of substitute decision-making to Chapter 1 from Chapter 3. In Japan, as no data are available regarding quality of life of patients who have undergone PEG, we added the perception of families of dementia patients who have undergone PEG instead; 26.7% of 33 families answered that they “wavered in judgment between ‘good’ and ‘not good’”, and 13.3% answered “not good” [8].

The original version of the decision aid was 36 pages in length. The Japanese version was condensed to 17 pages by increasing the number of sentences on each page. However, the contents of the decision aid were not changed from the original version, and the same illustrations were used after obtaining the developer’s consent.

### Evaluation of the decision aid

A before-and-after study was conducted to evaluate the tube feeding decision aid. A convenience sample of substitute decision makers who were at the point of deciding whether to place a PEG tube in older family members was recruited in three acute care hospitals and two mixed-care hospitals in Japan between April 2012 and January 2013. Physicians referred cases to the study. Inclusion criteria for patients were: age 65 years and older; severe cognitive impairment such that they could not make their own healthcare decisions; and placement of a PEG tube was being considered based on the clinical assessment of the healthcare team. Substitute decision makers had to be able to communicate in Japanese and consent to the study.

The pre- and post-questionnaires consisted of: (1) knowledge about swallowing problems, PEG tubes, risks and benefits of tube feeding, supportive care, and substitute decision-making; (2) predisposition to options; and (3) decisional conflict. The developer’s consent was obtained, and the pre- and post-questionnaires were translated into Japanese.

After obtaining informed consent, a researcher or research assistant administered the pre-questionnaire to the substitute decision maker. At this time the substitute decision maker did not receive information about PEG from the researcher/research assistant or physician. Then, the physician gave the substitute decision maker information about the patient’s condition and the reasons for considering tube feeding. The researcher or physician then gave the substitute decision maker information about tube feeding using the decision aid booklet. In the previous study, a self-administered, self-paced audio booklet was used as the decision aid. However, in Japan, because healthcare providers often provide information to patients and families orally and respond to their questions at that time, we used a paper booklet rather than a self-paced audio booklet. The researcher or physician helped the substitute decision maker to read the booklet and replied to the decision maker’s questions. The researcher or physician and substitute decision maker discussed the effects of performing PEG on quality of life. After working through the decision aid, the substitute decision maker decided whether to perform PEG and completed a post-questionnaire.

Knowledge was assessed in a multiple-choice format, and the proportion of questions answered correctly was compared before and after using the decision aid. Comfort with decision-making was assessed using the Decisional Conflict Scale [16], which was translated into Japanese with the developer’s consent. Based on the responses to 16 questions, this scale evaluates five domains of decisional conflict: (1) certainty regarding choices; (2) feeling informed; (3) feeling clear about values; (4) feeling supported in decision-making; and (5) quality of the decision. Each question is rated on a five-point Likert scale, with higher values representing greater decisional conflict. High conflict is indicated by an average score of 2.5 or more. Predisposition toward options was determined using a five-point learning scale that ranged from “in favor of” to “against” tube feeding.

The post-questionnaire also assessed acceptability by asking the participants whether they found the decision aid to be appropriate in length, clear, balanced, and helpful, and whether they would recommend it to others.

### Statistical analysis

Paired *t*-tests were used to compare subject’s knowledge and decisional conflict scores before and after using the decision aid.

## Results

**Figure 1:**
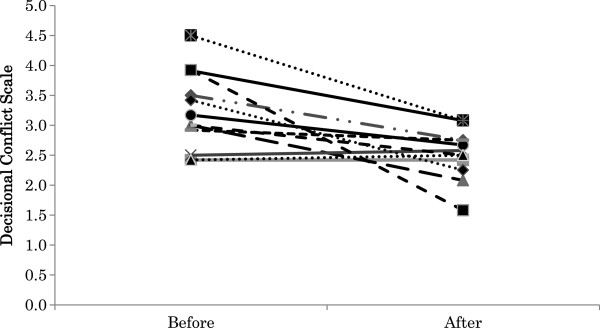
Decisional conflict scores. Comparison of mean decisional conflict score (y-axis) pre- and post-exposure to the tube feeding decision aid (3.24±1.37(SD) vs. 2.56±1.16(SD), P < .001).

**Figure 2:**
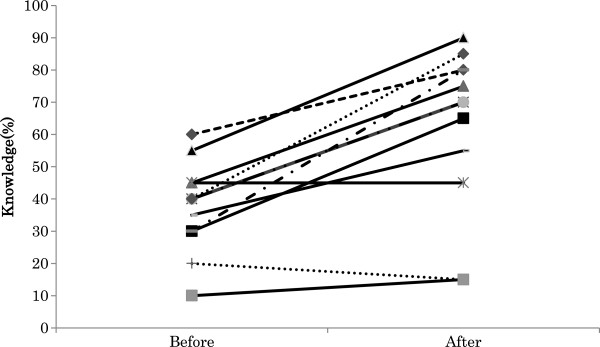
Knowledge scores. Comparison of mean percent of knowledge questions answered correctly (y-axis) pre- and post-exposure to the tube feeding decision aid (38.1%±13.5%(SD) vs. 64.6%±25.9%(SD), P < .001).

A total of 15 substitute decision makers were recruited, and 13 completed the before-and-after study. The mean age of the patients was 84.5±8.9(SD) years; seven were female. The diagnostic indications for PEG placement included cerebral infarction (n = 4), aspiration pneumonia (n = 4), pneumonia (n = 1), myeloma (n = 1), anorexia (n = 1), depression (n = 1), and symptomatic epilepsies (n = 1). Some substitute decision makers participated alone, and some participated with other persons, in which case the primary substitute decision maker completed the questionnaire. Substitute decision makers had the following relationships to the patients: son (n = 6), daughter (n = 2), daughter-in-law (n = 1), wife (n = 3), and niece (n = 1). The mean age of substitute decision makers was 62.8±10.0(SD) years; seven were male. The researcher provided information about tube feeding using the booklet and the decision aid to five of the 13 substitute decision makers, and the physician did so for the other eight substitute decision makers. The physicians provided information to substitute decision makers when the researchers were not able to travel to the hospitals. There was no difference in the content of the information provided by the researchers and physicians. The mean score for the Decisional Conflict Scale decreased significantly after exposure to the tube feeding decision aid compared with before (2.56±1.16(SD) vs. 3.24±1.37(SD), P < .01) (Figure 1). The mean score of “quality of the decision” after using the decision aid was 2.00±0.80(SD). This indicates that subjects became more comfortable with the decision-making process. The mean percentage of knowledge questions answered correctly by subjects was significantly greater after using the decision aid (64.6%±25.9%(SD) vs. 38.1%±13.5%(SD), P < .001) (Figure 2). The maximum of the difference between before and after was 50%. The minimum of the difference between before and after was 5%. Eleven substitute decision makers improved their knowledge scores; eight of 13 subjects increased more than 30%.

Two of 13 substitute decision makers were clearly in favor or slightly in favor of tube feeding at baseline. Seven of 13 substitute decision makers were clearly against or slightly against tube feeding at baseline. Four of 13 substitute decision makers were unsure. After using the decision aid, six of 13 substitute decision makers were clearly in favor or slightly in favor of tube feeding. Five of 13 substitute decision makers were clearly against or slightly against tube feeding. Two of 13 substitute decision makers were unsure. Substitute decision makers who were clearly against (n = 2) tube feeding at baseline did not change their preferences after using the decision aid. Of those who were unsure at baseline (n = 4), two were slightly in favor and two remained unsure after working through the aid. Of those who were slightly against at baseline (n = 3), two changed to slightly in favor and one changed to clearly against.

While working through the decision aid, the substitute decision makers asked questions; some asked about kind of facility patients can live in, and whether there are facilities that provide supportive care to patients who have not undergone PEG.

The decision aid was acceptable to the substitute decision makers. One subject did not answer the questions; the other 12 replied to all question items. All 12 subjects stated that they found it helpful; 11 of 12 subjects found that the decision aid was clear, and eight of 12 subjects found it appropriate in length. However, four of 12 subjects found it a little too long.

## Discussion

The decision aid significantly increased substitute decision makers’ knowledge and reduced their decisional conflict. Substitute decision makers found the decision aid helpful. It is very important that this decision aid, which has been proven to be effective in Canada, is also effective in Asia.

The results of the current study in Japan showed some differences compared with the previous study in Canada [14]. The mean age of substitute decision makers was older in Japan (62.8 vs. 56.5 years), and the percentage of knowledge questions answered correctly after using the decision aid was lower in the present study than in the previous study (62.1% vs. 84.0%). In addition, four of 12 subjects in the present study found the decision aid booklet to be a little too long. In Japan, substitute decision makers (patients’ families) have aged; therefore, it may be difficult for them to read long, detailed decision aid booklets. Thus, the contents of the booklet must be carefully selected, and the overall length of the aid must be shortened. In addition, the literacy of substitute decision makers may vary, so some substitute decision makers may have difficulty understanding written protocols for randomized controlled trials. We are currently considering ways to improve substitute decision makers’ understanding, such as adding an appendix to the end of the booklet and simplifying the language used in the booklet. From now on, a detailed healthcare provider-oriented version and a simple substitute decision maker-oriented version are needed. As compared with the previous study about the Decisional Conflict Scale, conflict was reduced significantly more in the present study. However, many subjects did not change or increase their score on the Decisional Conflict Scale after using the decision aid compared with the previous study (23.1% vs. 13%). The mean of the Decisional Conflict Scale after using the decision aid for subjects was still high (2.56). Thus, in Japan, the decision to place a loved one on tube feeding involves a high level of conflict. We need to consider the causes of this conflict and formulate approaches to reduce the conflict.

Several studies have identified a lack of communication between physicians and substitute decision makers. It was reported that 26.1% of Canadian decision makers and 10.4% of US decision makers for tube feeding patients with dementia did not discuss feeding tube insertion [17]. A five-state study in the US showed that 13.7% of substitute decision makers did not discuss with the physician, and 12.6% felt pressured by the physician to insert a feeding tube [9]. In Japan, family members of elderly patients with advanced dementia decided on PEG based on their trust in the physician and their desire to prolong the patients’ life. However, the family members wanted a sufficient explanation by a physician [18]. Another study in Japan found that, of 30 physicians, many decide to perform PEG before they explain the procedure to the family. When obtaining informed consent to perform the procedure from the family, many physicians are aware that such “informed consent” is not technically appropriate, because they lead the family to agree with the physician’s decision [19]. This requires the substitute decision maker to come to a decision in a situation of internal conflict without the benefit of adequate explanation from the physician. In such a situation, we think a decision aid booklet will become a tool that promotes communication between physicians and substitute decision makers. By sharing the principles of substitute decision-making between physicians and substitute decision makers via the booklet, emphasizing consideration of the previous wishes of the patient, both substitute decision makers and physicians can discuss the best option for patients. In this way, both patients and substitute decision makers can become involved in the decision. The physicians who used the booklet in the present study evaluated the booklet highly, and responded that they would use the booklet when providing information about tube feeding with regard to choosing whether to start tube feeding and considering the previous wishes of patients with their families.

The important point of substitute decision-making is to consider the wishes of the patient. The WHO prepared a report called “Better Palliative Care for Old People” in 2004 [20]. This report noted a lack of information and involvement in decision-making and the need for involvement in decision-making. In Japan, it is difficult to talk about death with elderly people, and many elderly people prefer to entrust decisions about the end of life to medical staff and family members. This makes it difficult to determine elderly patients’ wishes about end-of-life care in advance. The present decision aid booklet describes the first step in substitute decision-making as considering the previous wishes of the patient. We think that if substitute decision makers can consider the previous wishes of the patient, the patient’s intentions can be respected and their dignity can be protected, and substitute decision makers can be confident in their decisions. However, families rarely ask about the patient’s intentions in advance, and families experience great confusion and unease in the process of decision-making [21]. In Japan, the use of advance directives is inadequate; only 2% of workers in nursing homes know the exact meaning of an advance directive [22]. Another study of ordinary persons of middle or advanced age found that most of those who answered did not desire an advance directive, since they preferred that their physician or family just decide the treatment policy according to the situation at that time [23]. It is thus necessary to have an appropriate legal framework and education about advance directives in Japan.

In Japan, when patients transition from acute hospitals to non-acute hospitals or nursing homes, they are often required to undergo PEG in order to reduce the burden on staff who help patients with swallowing difficulty to eat. Non-acute hospitals and nursing homes often have staff shortages. If there is no possible place of care other than a non-acute hospital or nursing home, then there actually may be no choice other than to undergo PEG. This may partly explain why many people are in favor of PEG. This is a serious problem that affects the judgment of substitute decision makers.

The present research has several important limitations. First, because the present study included a small number of subjects, the generalization of the results is limited. Second, the design of this research was a before-and-after study. A randomized controlled trial is needed to show higher validity, but few American studies have examined decision-making about feeding options, goals-of-care, and cardiopulmonary resuscitation using a randomized controlled trial design [24-26]. Third, the present study focused on decision-making at the time of deciding whether to introduce tube feeding. Although the degree of satisfaction of the family immediately after the decision aid was measured, the family’s satisfaction with their decision was not measured after a period of continuous tube feeding. It is important that the degree of satisfaction of family members who made decisions after using a decision aid be evaluated on an ongoing basis, so that the long-term appropriateness of the decision aid may be evaluated. Third, the setting of the present study was an acute care hospital and a mixed care hospital. The place of medical treatment in Japan is increasingly shifting to the home. Thus, it is also necessary to evaluate use of the decision aid for patients at home.

## Conclusions

The present decision aid facilitated the decision-making process of substitute decision makers for cognitively impaired older patients in Japan regarding long-term tube feeding by decreasing decisional conflict and increasing knowledge. This decision aid booklet is expected to promote communication between physicians and substitute decision makers. A randomized controlled trial design is needed to examine decision-making further with higher validity, and continuing research dealing with substitute decision makers who made decisions about tube feeding is needed to clarify the long-term appropriateness of decisions reached using the decision aid.

## Competing interests

This work was supported by MEXT KAKENHI (Grant Number 22890189).

## Authors’ contributions

YK was involved in all aspects of the research, including study design, informant recruitment, data analysis, and manuscript preparation. KN’s role in the study included assistance with study design, supervision of data collection, data analysis, and preparation of the manuscript. Both authors read and approved the final manuscript.

## Pre-publication history

The pre-publication history for this paper can be accessed here:

http://www.biomedcentral.com/1471-2318/14/16/prepub
